# Factors associated with bone mineral content in adults: a population-based study

**DOI:** 10.31744/einstein_journal/2020AO4694

**Published:** 2019-10-22

**Authors:** Kátia Josiany Segheto, Leidjaira Lopes Juvanhol, Cristiane Junqueira de Carvalho, Danielle Cristina Guimarães da Silva, Adriana Maria Kakehasi, Giana Zarbato Longo

**Affiliations:** 1 Universidade Federal de Viçosa, Viçosa, MG, Brazil.; 2 Universidade Federal do Oeste da Bahia, Barreiras, BA, Brazil.; 3 Universidade Federal de Minas Gerais, Belo Horizonte, MG, Brazil.

**Keywords:** Bone density, Adult, Risk factors, Densitometry, Epidemiology

## Abstract

**Objective:**

To determine the association among bone mineral content, sociodemographic, anthropometric and behavioral factors, and health status of Brazilian adults.

**Methods:**

This was a cross-sectional, population-based study including 701 individuals from both sexes aged between 20 and 59 years. DEXA was used to evaluate dependent variable. The associations were evaluated using linear regression models stratified by sex.

**Results:**

When mean bone mineral content values were compared, we found significant differences related to sex and all the independent variables evaluated. In the adjusted models, we identified an inverse association between bone mineral content and age in both sexes. Among men, to be overweight and/or obese, be highly educated, and have almost sufficiency of 25(OH)D were associated with higher bone mineral content values. On the other hand, among women, to be non-white skin color, overweight and/or obese were associated with better bone health. The main factors associated with low total bone mineral density were advanced age, white skin color, low level of formal education, eutrophy, and 25(OH)D deficiency.

**Conclusion:**

Our results may help to identify adults who are at higher risk, and these findings should be used as guidelines for prevention and early diagnosis.

## INTRODUCTION

According to the World Health Organization (WHO), life expectancy has been increasing gradually, and according to recent data released, the world age projection for people who were born in 2015 is around 71 years for women and 69 for men. This population aging framework causes an important impact on public health, with significant increase in mortality by chronic non-communicable diseases, such as those related to low bone mineral density (BMD).^1^

To understand the maturation process and consequent decline of bone health throughout development is important, mainly in the adult phase, since this osteometabolic deterioration alters the microarchitecture of tissue, which can also increase the probability of fracture occurrence.^2^

Diseases related to low BMD are multifactorial, and the associations have not yet been well established in adults. Concerning individuals’ age and sex,^3 , 4^ bone metabolism is also influenced by genetics,^5^ hormone replacement, use of medication,^6 , 7^ sunlight exposure deficiency and vitamin D insufficiency,^8 , 9^ body composition,^10 , 11^ smoking,^12^ alcoholism,^13^ physical activities,^14^ and level of formal education.^15 , 16^ However, most studies have evaluated the association BMD, and these factors in older individuals isolately have not been considered in studies as well as the inter-relationship among BMD and behavioral and health conditions.^17 , 18^ Studies evaluating bone mineral content (BMC) in adults are scarce.^19 , 20^ Although BMC is not often used in clinical practice to assess adult bone health, the use of total BMD and its respective association factors is important, because BMC could be another important evaluation parameter. Moreover, it allows us developing strategies for early diagnosis and prevention of severe bone loss.

## OBJECTIVE

To verify the association between bone mineral content and sociodemographic, anthropometric, behavioral and health conditions in Brazilian adults.

## METHODS

This is a cross-sectional, population-based study carried out from 2012 to 2014 including individuals of both sexes. Participants’ age ranged from 20 to 59 years, and they were residents of the urban area of the municipality of Viçosa (MG), Brazil.

Sampling process was done by two-stage conglomerates. The first-stage units were composed by the census sectors, and the second-stage units, by households. Once the census sector and the blocks were selected, the steps of the study’s procedures were informed to the residents who met the inclusion criteria.

The following parameters were used to calculate the sample: unknown prevalence of 50% for the outcome (BMC expressed in g); effect of the conglomerate design, which is estimated at 1.50; percentage loss estimated at 10%, and 10% more to control confounding factors. For this reason, it was estimated that a minimum of 692 individuals were required to carry out this study. Dividing the value by number of drawn census sectors 23 people were required for the investigation in each sector, corresponding to every 3 households visited.^21^ The final sample of this study consisted of 701 individuals.

Bone mineral content was evaluated using a Lunar Prodigy Advance DXA System Dual energy X-ray absorptriometry (DEXA) device (GE Healthcare, Brazil). This is a high technological imaging system with low radiation, good reproducibility and non-invasive, which is considered as gold standard for bone health assessment, and is recommended by the WHO.^22^ The accuracy and safety of the DXA device were assessed before tests were conducted, with the individual in the dorsal decubitus position, and by mapping individuals’ total body bone density. The results were compiled by a medical specialist and presented to the volunteers as a report. Absolute values of BMC (g) were considered for statistical analyses.

The volunteers in the households responded to a semi-structured questionnaire applied by trained interviewers to collect information on sociodemographic, health, and behavioral conditions. The variables in this study were sex (man and woman), age (stratified into 20 to 29, 30 to 39, 40 to 49 and 50 to 59 age groups), skin color (white and non-white), years of formal education (zero to 4, 5 to 8, 9 to 11 and ≥12 years of study), menopause, use of contraceptives and hormone replacement therapy (all of them categorized into yes and no), alcohol consumption (divided into three categories, according to the weekly consumption of alcoholic beverages, which was zero; 1 to 7 and ≥8 drinks), and smoking (non-smokers, former-smokers and smokers).

Data referring to physical activity level (PAL) and body mass index (BMI) were collected in the laboratory. Physical activity level was measured using the International Physical Activity Questionnaire (IPAQ), version 6, long format, validated to be applied to Brazilian young adults population.^23^ The PAL score was calculated by summing the time spent with physical activity of moderate intensity (including walking) and vigorous intensity, which was obtained by the time spent with vigorous physical activity multiplied by two [(PAL = moderate physical activity + (vigorous PA × 2)].^24^ From this result, the PAL of the evaluated individuals was calculated according to domain 4 in the IPAQ referring to recreational, sports, exercise, and physical leisure activities.^24^ The PAL was categorized into irregularly active (50 minutes per week) and physically active (>150 minutes per week).^25^ Body mas index was calculated by the formula [weight(kg)/height^2^ (m^2^ )] and categorized into eutrophic (≤24.9kg/m^2^ ), overweight (25.0kg/m^2^ to 29.9kg/m^2^ ), and obese (≥30.0kg/m^2^ ).^26^

The total body mass (kg) for the BMI calculation was weighed on a digital scale (TANITA, model BC-554) with the individuals wearing least as possible clothing and barefoot. Height (cm) was measured directly using a wall stadiometer with standing individuals, barefoot, heels together, touching the measurement bar and with arms hanging naturally down along side the body.

Blood samples, which required a 12-period of fasting, were collected from 7 a.m. to 10 a.m in a laboratory using a disposable vacuum collection system. The 25-hydroxyvitamin D [25(OH)D] was evaluated by chemiluminescence using the ARCHITECT 25(OH)D reagents (Abbott Architect I Instrument, Illinois, USA).^27^ The 25(OH)D status was determined according to reference values: sufficient (≥30.0ng/mL), insufficient (21.0ng/mL to 29.9ng/mL), and deficient (<20.9ng/mL).^27^ Although there is a new proposal for the 25(OH)D reference intervals to the Brazilian population,^28^ these values have been recently presented, and therefore, we opted to use the current classification during our study data collection.

The results were entered twice into an EpiData database, version 3.1. After verifying the data consistency, analyses were performed using the Stata 13.1 statistical package. The analysis was weighed based on sex, age, and formal education. Weights were determined by ratio between the proportions of individuals in the study’s sample.^21^ Proportions, means, and the respective confidence intervals of the variables studied for the overall sample and the sample stratified by sex were calculated, considering a 95% confidence level (95%CI). The statistical difference between the evaluated samples was verified by comparing confidence intervals, and those not showing overlap were significant. The normal dependent variable BMC was assessed by the Shapiro-Wilk test and histograms, indicating normal distribution. The associations between the BMC and the independent variables were investigated by linear regression, using p<0.25 as an entry criterion into the multiple model, and an overall significance level of α=0.05.

This research was approved by the Ethics Committee Research involving human at the *Universidade Federal de Viçosa* (UFV) (number 02/2013/CEP/12.07.2013) according to the principles of the Helsinki Declaration and to the resolution of the National Health Council 466/2012. All the volunteers who agreed to participate signed an Informed Consent Form.

## RESULTS

Of the total 701 individuals included in the study, 50.3% were women, 26.2% were aged between 30 and 39 years, 61.4% reported themselves as non-white, and 42.8% were highly educated having completed more than 12 years of formal education. The sample presented 50.3% of eutrophic individuals, 72.6% of irregularly active individuals, 65.1% of non-smokers, and 45.9% did not consume alcoholic beverages. Regarding health condition, 49.0% of the evaluated patients presented 25(OH)D sufficiency. Of the total number of evaluated women, 64.7% were non-menopausal, 96.4% did not take hormone replacement, and 81.0% used contraceptives. In relation to comparison of proportions between sexes, we found that 62.0% of women did not consume alcoholic beverages and 23.2% of men consumed eight or more drinks. For this reason, differences in these categories were significant. In addition, men showed a lower proportion of 25(OH)D deficiencies than women, whereas this difference was also significant ( Table 1 ).

**Table 1:** Characterization of the study population, according to sociodemographic, behavioral and health condition variables, stratified by sex

Variables	Total		Men		Women	
		
	%	CI95%	%	CI95%	%	CI95%
Sex	-	-	49.7	45.2-54.2	50.3	45.8-54.8
Age range, years						
20-29	24.3	17.4-33.4	28.9	19.1-41.2	19.8	13.8-27.6
30-39	26.2	21.8-31.0	26.6	20.4-34.0	25.7	21.0-31.0
40-49	24.0	19.1-29.7	21.9	15.2-30.4	26.2	20.6-32.5
50-59	25.5	20.1-31.7	22.6	16.1-30.8	28.3	22.2-35.4
Skin color						
White	38.6	31.7-45.9	42.0	32.9-51.7	35.2	28.6-42.3
Non-white	61.4	54.1-68.3	58.0	48.3-67.1	64.8	57.7-71.3
Education, years						
0-4	20.1	12.8-29.1	16.7	7.7-32.8	23.4	16.2-32.5
5-8	16.3	11.9-21.7	15.7	10.4-23.0	16.7	11.9-23.0
9-11	20.8	17.4-24.7	20.0	14.1-27.3	21.7	18.7-25.1
≥12	42.8	30.8-55.7	47.6	32.6-63.1	38.2	27.1-50.5
Nutritional status						
Eutrophic	50.3	43.2-57.4	47.7	38.7-56.8	53.0	43.5-62.2
Overweight	33.4	28.3-38.8	39.6	31.6-48.2	27.2	22.1-33.0
Obesity	16.3	12.7-20.5	12.7	8.8-17.9	19.8	14.6-26.1
PAL						
IA	72.6	66.8-78.4	73.13	63.5-80.9	72.16	65.9-77.6
PA	27.4	21.6-33.9	26.86	19.0-36.5	27.84	22.4-34.0
Smoking						
Non-smoker	65.1	57.9-71.6	61.9	50.5-72.1	68.3	60.7-75.1
Smoker	13.0	9.9-16.5	15.2	9.8-22.7	10.7	7.8-14.7
Former-smoker	21.9	15.8-29.5	22.9	13.5-36.3	21.0	15.3-28.0
Alcohol consumption, drinks/week						
0	45.9	41.7-50.2	29.8	23.8-36.6*	62.0	55.5-68.0*
1-7	40.3	35.1-45.7	47.0	38.1-55.0	33.7	28.1-39.8
8 or more	13.8	10.0-18.4	23.2	17.4-30.3*	4.3	2.5-7.5*
Menopause					64.7	56.9-71.7
No	-	-	-	-	35.3	28.3-43.1
Yes	-	-	-	-		
Hormone replacement therapy						
No	-	-	-	-	96.4	94.3-97.7
Yes	-	-	-	-	3.6	2.3-05.7
Contraceptive						
No	-	-	-	-	19.0	15.4-23.1
Yes	-	-	-	-	81.0	76.8-84.6
25(OH)D						
Sufficient	49.0	42.5-55.5	55.7	47.4-63.6	42.4	35.0-50.0
Insufficient	37.5	32.7-42.6	35.3	28.8-42.3	39.8	34.7-45.0
Deficient	13.5	10.5-17.1	9.0	6.3-12.7*	17.8	13.1-23.8*

* Statistically significant difference, p<0.05, for comparison between sex. CI95%: confidence interval of 95%; PAL: physical activity level; IA: irregularly active; PA: physically active; 25(OH)D: 25- hydroxyvitamin D.

The mean BMC values were significantly higher among men. Better bone health was observed among overweight and obese individuals compared to eutrophic from both sexes. A higher mean BMC was only found among younger women (20 to 29-year group) in comparison with older women (50 to 59-year group) who self-reported themselves as non-white, not being in menopause, and not using contraceptives ( Table 2 ).

**Table 2:** Bone mineral content, according to sociodemographic, behavioral and health condition variables, stratified by sex

Variables	Men		Women	
	
	Mean	CI95%	Mean	CI95%
Sex	3.040	2.972-3.109	2.323	2.284-2.362
Age range. years				
20-29	3.122	3.025-3.219	2.415	2.336-2.495*
30-39	3.042	2.929-3.154	2.408	2.331-2.486
40-49	3.031	2.894-3.169	2.359	2.297-2.421
50-59	2.944	2.816-3.073	2.148	2.050-2.247*
Skin color				
White	2.985	2.895-3.075	2.245	2.187-2.302*
Non-white	3.081	2.976-3.186	2.365	2.306-2.425*
Education, years				
0-4	2.878	2.716-3.040	2.223	2.102-2.345
5-8	2.968	2.830-3.106	2.373	2.265-2.480
9-11	3.094	2.950-3.238	2.312	2.230-2.394
≥12	3.101	3.006-3.196	2.369	2.313-2.425
Nutritional status				
Eutrophic	2.835	2.744-2.927*^†^	2.225	2.183-2.267*^†^
Overweight	3.175	3.043-3.307*	2.396	2.313-2.479*
Obesity	3.423	3.307-3.539^†^	2.499	2.388-2.609^†^
PAL				
IA	2.992	2.923-3.060	2.319	2.268-2.369
PA	3.174	3.032-3.316	2.333	2.264-2.401
Smoking				
Non-smokers	3.041	2.952-3.130	2.328	2.285-2.371
Smoker	3.105	2.994-3.217	2.302	2.190-2.414
Former-smoker	2.996	2.894-3.098	2.317	2.224-2.410
Alcohol consumption, drinks/week				
0	2.999	2.866-3.133	2.319	2.270-2.368
1-7	3.038	2.945-3.131	2.322	2.251-2.393
8 or more	3.098	2.953-3.243	2.390	2.195-2.586
Menopause				
No	-	-	2.396	2.336-2.455
Yes	-	-	2.190	2.103-2.277
Hormone replacement therapy				
No	-	-	2.323	2.283-2.363
Yes	-	-	2.309	2.152-2.466
Contraceptive				
No	-	-	2.294	2.243-2.346
Yes	-	-	2.429	2.346-2.513
25(OH)D				
Sufficient	3.100	3.019-3.181	2.339	2.283-2.395
Insufficient	2.994	2.874-3.113	2.298	2.241-2.356
Deficient	2.861	2.693-3.028	2.338	2.229-2.446

*^†^ Equal symbols indicate a statistically significant difference (p<0.05) for the comparison between the averages of bone mineral content according to the categories of the variables within each sex. CI95%: 95% confidence interval; BMC: bone mineral content; PAL: physical activity level; IA: irregularly active; PA: physically active; 25(OH)D: 25-Hydroxyvitamin D.

Tables 3 and 4 show unadjusted and adjusted associations between BMC and independent variables according to sex. Bone mineral content decreased with age in both sexes (men: p=0.008; women: p<0.001), and this association is more pronounced after adjustment (men: p=0.007; women: p<0.001). The variable nutritional status was also associated with BMC in the adjusted models for both sexes (men: p<0.001; women: p<0.001), which was a positive association. We observed a significant association between high level of formal education and increased BMC only observed among men (p=0.003), and 25(OH)D deficiency that was significantly associated with low BMC (p<0.001). Bone mineral content was also significantly higher among women who self-reported to be non-white (p=0.010).

**Table 3:** Coefficients of simple and multiple linear regressions, confidence intervals and p-value for bone mineral content in men

BMC (g)	β	CI95%	p value*	βaj	CI95%	p value^†^
Age range, years			0.008			0.007
20-29	-	-		-	-	
30-39	-0.08	-0.22-0.06		-0.03	-0.16-0.08	
40-49	-0.09	-0.24-0.06		-0.14	-0.28- -0.06	
50-59	-0.17	-0.31- -0.03		-0.17	-0.31- -0.03	
Education, years			0.011			0.003
0-4	-	-				
5-8	0.09	-0.12-0.30		0.11	-0.11-0.33	
9-11	0.21	-0.02-0.45		0.20	-0.03-0.43	
≥12	0.22	0.03-0.41		0.27	0.07-0.48	
Nutritional status			<0.001			<0.001
Eutrophic	-	-		-	-	
Overweight	0.33	0.18-0.49		0.41	0.28-0.54	
Obesity	0.58	0.43-0.74		0.67	0.5-0.85	
25(OH)D			0.005			<0.001
Sufficient	-	-		-	-	
Insufficient	-0.10	-0.23-0.02		-0.14	-0.26 -0.02	
Deficient	-0.23	-0.4- -0.07		-0.32	-0.45- -0.18	

* Simple linear regression at significance level p<0.25; ^†^ multiple linear regression at significance level p<0.05. β: simple linear regression; CI95%: 95% of confidence interval; βAj: ß value adjusted to covariables; BMC: bone mineral content; 25(OH)D: 25-hydroxyvitamin D.

**Table 4:** Coefficients of simple and multiple linear regressions, confidence intervals and p-value for bone mineral content in women

BMC (g)	β	CI95%	p value*	βaj	CI95%	p value^†^
Age range, years			<0.001			<0.001
20-29	-	-		-	-	
30-39	-0.07	-0.10-0.90		-0.08	-0.18- 0.13	
40-49	-0.05	-0.16-0.05		-0.17	-0.27 -0.65	
50-59	-0.02	-0.04-0.01		-0.38	-0.53- -0.24	
Skin color			0.013			0.010
White	-	-				
Non-white	0.12	0.02-0.21		0.91	0.24-0.15	
Nutritional status			<0.001			<0.001
Eutrophic	-	-		-	-	
Overweight	0.17	0.08-0.25		0.22	0.14-0.31	
Obesity	0.73	0.16-0.17		0.34	0.26-0.43	

* Simple linear regression at significance level p<0.25; ^†^ multiple linear regression at significance level p<0.05. β: simple linear regression; CI95%: 95% of confidence interval; βAj: β value adjusted to covariables; BMC: bone mineral content; 25(OH)D: 25-hydroxyvitamin D.

## DISCUSSION

In our study, the mean BMC in men were significantly higher than in women. In addition, related to the difference between sexes we found that main factors associated with BMC were age and nutritional status. Moreover, an association between formal education and 25(OH)D was observed in men. Among women, this association was related with skin color.

Our findings of the association between sex and bone health for women presented significantly lower mean BMC which is consistent with other studies.^3 , 4^ This difference is primarily related to hormonal issues^29^ and it intensifies after menopause, when there is a significant reduction in estrogen levels.

In addition, the loss of BMC during adulthood may also be related to environmental^30^ and genetic factors,^5^ as well as a long-term use of contraceptives.^18^

Our results in terms of age corroborate with other studies.^3 , 4^ We found that as age increases, the balance between bone formation and absorption changes and bone mineral progressively decreases. This loss is more pronounced after peak bone mass, which occurs around 30 years of age, and then gradually declines, and this intensifies as age advances.^31^ Therefore, balanced bone metabolism, during adulthood, will provide adequate maintenance to bone health and may be an important factor in controlling bone mass loss and avoid the risk of fractures in older individuals.

Therefore, after the period of peak bone mass, preventive procedures should be started to avoid a marked bone decline in more advanced ages.

In this sense, regular physical activity is an important factor that can act as a non-pharmacological alternative to prevent the consequences resulting from low bone mineral.^14^ Although no significant differences were observed related with this variable in our study, a high mean BMC were found between physically active individuals. Thus, there is a need for further investigations relating bone mineral to individuals’ PAL history throughout their development.

In this study, nutritional status (overweight and obesity) was a factor positively associated with BMC for both men and women. This result is consistent with findings reported by another study with young adults,^10^ and this positive association can be explained by the greater mechanical overload among individuals with excessive weight. Such findings reinforce the theory that bone tissue needs to undergo mechanical stress for bone formation and remodeling, thus maintaining active bone metabolism.^11^ On the other hand, it should be reinforced that there are hormonal consequences from high body weight that may harm bone health such as type 2 diabetes, metabolic syndrome, insulin resistance, and hyperglycemia, among others.^32^

Positive associations between level of formal education and BMC were also identified among men, indicating that the longer study time the better the bone health. This data corroborate with other studies including adults.^15 , 16^ This positive association supposes that for diseases related to bone health, the higher the education level the greater the access to information and knowledge about preventive care and attitudes such as regular physically active and balanced diet, which may positively reflect bone health in more advanced ages.^15 , 16^

Another finding of our study was the direct relationship between 25(OH)D sufficiency and BMC among men, which is similar findings reported by other studies.^8 , 9^ Given the importance of 25(OH)D for bone metabolism (absorption and maintenance of calcium and phosphate blood levels),^27^ if during adulthood the metabolism of this vitamin is balanced, it may maintain adequate bone health during this development stage and may be an important factor to control BMC loss and avoid the risk of fractures in more advanced ages.

We identified skin color as an association factor for women, because non-white women were positively associated with BMC. A great variation in the concentrations of 25(OH)D exist according to skin color. As reported by other studies, non-white individuals show insufficient 25(OH)D, but they have better bone health and lower risk for fracture.^33^

This association requires further studies because there are still gaps in the published literature regarding skin color, 25(OH)D metabolism, and bone health among different populations.

The positive aspects of this study are its population-based approach, the use of validated instruments, the standardization of data collection procedures, and adoption of quality control strategies. In addition, our study evaluated risk factors for low bone health in healthy men, which is different from the majority of studies that evaluated postmenopausal women, individuals with older age, and relationship of these groups with a specific disease.

Considering that peak bone mass occurs around the age 30 in both sexes and that a gradual decline occurs thereafter, as previously mentioned, we also evaluated if factors associated with BMC differ in individuals younger and older than 30 years. However, no significant changes were observed in relation to the magnitude, direction and statistical significance of the associations in the stratified analyzes.

Other issue considered in our analysis was a possible limitation of BMI as a method to evaluate overweight and obesity. For this reason, to introduce the fat percentage we considered in the final model categories based on three-fold protocol^34 , 35^ that replaced the BMI variable. In this analysis, we identified that the final model remained unchanged, and results presented the same association factors both for men and for women. This study evaluated the factors associated with BMC, and results are presented using the BMI since it is a practical, easy-to-apply and low-cost measure recommended by the WHO^26^ for nutritional status classification in healthy overweight or underweight individuals.

This is a cross-sectional study and, as such, the identified associations could not be interpreted as a causal relationship. Our results may contribute to better understand bone health in adults, as well as the development of prevention strategies related to bone health.

## CONCLUSION

The findings of this study indicate that the main factors associated with low bone mineral content were advanced age, white skin color, low formal education, eutrophy and 25(OH)D deficiency. Differences were observed between men and women. Adult patients with such characteristics should be considered and advised about prevention strategies and early diagnosis. There is need to further the understandings about the behavioral parameters such as physical activity level, smoking habit, and alcoholism because there are still gaps in the published literature regarding association of these parameters with bone health.
