# Supplementary material for: Factors associated with bone mineral content in adults: a population-based study
Source: Einstein (Sao Paulo). 2019 Oct 22;18:eAO4694. doi: 10.31744/einstein_journal/2020AO4694 (PMC6896653; doi:10.31744/einstein_journal/2020AO4694)
Supplement: Translation

# Translation: Fatores associados ao conteúdo mineral ósseo em adultos: estudo de base populacional

**Authors:** Kátia Josiany Segheto, Leidjaira Lopes Juvanhol, Cristiane Junqueira de Carvalho, Danielle Cristina Guimarães da Silva, Adriana Maria Kakehasi, Giana Zarbato Longo

**Affiliations:** 1 Universidade Federal de Viçosa, Viçosa, MG, Brasil.; 2 Universidade Federal do Oeste da Bahia, Barreiras, BA, Brasil.; 3 Universidade Federal de Minas Gerais, Belo Horizonte, MG, Brasil.

**Keywords:** Densidade óssea, Adulto, Fatores de risco, Densitometria, Epidemiologia

## Abstract

**Objetivo:**

Verificar a associação entre o conteúdo mineral ósseo e fatores sociodemográficos, antropométricos, comportamentais e condições de saúde em adultos brasileiros.

**Métodos:**

Estudo transversal, de base populacional, realizado com 701 indivíduos de ambos os sexos, com idade entre 20 e 59 anos. A variável dependente foi avaliada por DEXA. As associações foram avaliadas por modelos de regressão linear estratificados baseados no sexo dos indivíduos.

**Resultados:**

Quando comparados os valores médios do conteúdo mineral ósseo, observamos diferença estatisticamente significante em relação aos sexos e para todas as variáveis independentes avaliadas. Nos modelos ajustados, identificamos associação inversa entre o conteúdo mineral ósseo e a idade em ambos os sexos. Entre os homens, sobrepeso e obesidade, alta escolaridade e suficiência de 25(OH)D foram associados a maiores valores de conteúdo mineral ósseo. Entre as mulheres, por sua vez, cor da pele não branca, sobrepeso e obesidade foram associados a melhor saúde óssea. Os principais fatores associados à baixa massa óssea total foram idade avançada, cor da pele branca, baixa escolaridade, eutrofia e deficiência de 25(OH)D.

**Conclusão:**

Esses resultados podem auxiliar na identificação de adultos com maior risco e que devem ser alvo de medidas de prevenção e diagnóstico precoce.

## INTRODUÇÃO

De acordo com a Organização Mundial da Saúde (OMS), a expectativa de vida tem aumentado gradualmente e, com base nos dados divulgados recentemente, a expectativa mundial em relação à idade para pessoas nascidas em 2015 é de 71 anos para mulheres e 69 para homens. Esse quadro de envelhecimento populacional causa impacto importante na saúde pública, com aumento significativo da mortalidade por doenças crônicas não transmissíveis, como as relacionadas à baixa densidade mineral óssea (DMO).^1^

É importante entender o processo de maturação e o consequente declínio da saúde óssea ao longo do desenvolvimento, principalmente na fase adulta, uma vez que essa deterioração osteometabólica altera a microarquitetura do tecido e também pode aumentar a probabilidade de ocorrência de fraturas.^2^

Doenças relacionadas à baixa DMO são multifatoriais, e ainda não há associações bem estabelecidas em pacientes adultos. Sobre a idade e o sexo dos indivíduos,^3 , 4^ tem sido apontado que o metabolismo ósseo também é influenciado por genética,^5^ reposição hormonal, uso de medicamentos,^6 , 7^ deficiência de exposição à luz solar e insuficiência de vitamina D,^8 , 9^ composição corporal,^10 , 11^ tabagismo,^12^ alcoolismo,^13^ atividade física^14^ e nível educacional.^15 , 16^ Entretanto, a maioria dos estudos tem avaliado a associação entre DMO, e fatores associados em indivíduos mais velhos e mulheres menopausadas, essencialmente.^17 , 18^

Estudos avaliando o conteúdo mineral ósseo (CMO) em adultos são escassos.^19 , 20^ Embora o CMO não seja usado com frequência na prática clínica para avaliar a saúde óssea em adultos, o uso de DMO total e seus respectivos fatores é importante, pois o CMO consiste em parâmetro fundamental de avaliação. Além disso, a avaliação desse parâmetro permite desenvolver estratégias para o diagnóstico precoce e prevenção da perda óssea grave.

## OBJETIVO

Verificar a associação entre o conteúdo mineral ósseo e as condições sociodemográficas, antropométricas, comportamentais e de saúde em adultos brasileiros.

## MÉTODOS

Trata-se de estudo transversal, de base populacional, conduzido de 2012 a 2014, incluindo indivíduos de ambos os sexos. A idade dos participantes variou de 20 a 59 anos, e todos eram moradores de área urbana do município de Viçosa (MG).

O processo de amostragem foi feito por conglomerados em dois estágios. As unidades de primeiro estágio foram compostas pelos setores censitários, e as unidades de segundo estágio, por domicílios. Uma vez que o setor censitário e os blocos foram sorteados, os procedimentos da pesquisa foram informados aos moradores, que preencheram os critérios de inclusão.

Os seguintes parâmetros foram utilizados para calcular a amostra: prevalência desconhecida de 50% para o desfecho (CMO expresso em g); e efeito do desenho de conglomerado estimado em 1,50, percentagem de perda estimada em 10% e consideração de mais 10%, para controle de fatores de confusão. Assim, estimou-se um mínimo de 692 indivíduos para realização do estudo. Dividindo o valor pelo número de setores censitários sorteados, foram necessárias 23 pessoas para a investigação em cada setor, o que foi alcançado a cada 3 domicílios visitados.^21^ A amostra final foi composta por 701 indivíduos.

O CMO foi avaliado usando o dispositivo *Lunar Prodigy Advance* Sistema DXA de absorciometria por raios X de dupla energia (DEXA; GE Healthcare, Brasil). Trata-se de um sistema de imagem de alta tecnologia com baixa radiação, boa reprodutibilidade e não invasivo, considerado padrão-ouro para avaliação da saúde óssea e recomendado pela OMS.^22^ A precisão e a segurança do dispositivo DEXA foram avaliadas antes, e os testes foram realizados com o indivíduo em decúbito dorsal e por mapeamento da densidade óssea total dos indivíduos. Os resultados foram compilados por profissional médico e apresentados aos voluntários em formato de relatório. Os valores absolutos de CMO (g) foram adotados para análises estatísticas.

Os voluntários dos domicílios responderam um questionário semiestruturado aplicado por entrevistadores treinados para coletar informações sobre condições sociodemográficas, de saúde e comportamentais. As variáveis desse estudo foram sexo (homem e mulher), idade (estratificada em 20 a 29, 30 a 39, 40 a 49 e 50 a 59 anos), cor da pele (branca e não branca), nível educacional (zero a 4, 5 a 8, 9 a 11 e> 12 anos de estudo), menopausa, uso de contraceptivos e terapia de reposição hormonal (sim e não), consumo de álcool (dividido em três categorias, de acordo ao consumo semanal de bebidas alcoólicas, que foi de zero, 1 a 7 e >8 bebidas) e tabagismo (não fumantes, ex-fumantes e fumantes).

Dados referentes ao nível de atividade física (NAF) e índice de massa corporal (IMC) foram coletados em laboratório. O NAF foi medido por meio do *International Physical Activity Questionnaire* (IPAQ), versão 6 completa, validado para ser aplicado à população brasileira adulta jovem.^23^ O escore do NAF foi calculado pela soma do tempo gasto com atividade física de intensidade moderada (incluindo caminhada) e intensidade vigorosa, obtida pelo tempo gasto com atividade física vigorosa multiplicado por dois [(NAF = atividade física + moderada + atividade física × 2 vigorosa).^24^ A partir desse resultado, o NAF dos indivíduos avaliados foi calculado de acordo com domínio 4 no IPAQ, referente às atividades recreativas, esportivas e exercício físico.^24^ O NAF foi categorizado em irregularmente ativo (<150 minutos por semana) e fisicamente ativo (>150 minutos por semana).^25^ O IMC foi calculado pela fórmula [peso (kg)/ altura^2^ (m^2^ )] e categorizado em eutrófico (<24,9kg/m^2^ ), sobrepeso (25,0kg/m^2^ a 29,9kg/m^2^ ) e obeso (>30,0kg/m^2^ ).^26^

A massa corporal total (kg) para o cálculo do IMC foi obtida por pesagem em balança digital (TANITA, modelo BC-554) com os indivíduos usando o mínimo possível de roupas e descalços. A altura (cm) foi medida diretamente utilizando estadiômetro na parede com indivíduos em pé, descalços, com os calcanhares juntos, tocando a barra de medição e com os braços estendidos naturalmente ao longo do corpo.

As amostras de sangue, que necessitaram de 12 horas de jejum, foram coletadas das 7h às 10h, em laboratório, com uso de sistema descartável de coleta a vácuo. A 25-hidroxivitamina D (25(OH)D) foi avaliada por quimiluminescência com reagentes ARCHITECT 25(OH)D (Abbott Architect I Instrument, Illinois, USA).^27^ O status da 25(OH)D foi determinado de acordo com os valores de referência: suficiente se >30,0ng/mL, insuficiente se 21,0ng/mL a 29,9ng/mL, e deficiente se <20,9ng/mL.^27^ Embora exista nova proposta para os intervalos de referência da 25(OH)D na população brasileira,^28^ esses valores foram recentemente apresentados, e optou-se pela classificação atual durante a coleta de dados do estudo.

Os resultados foram incluídos duas vezes no banco de dados EpiData, versão 3.1. Após verificação da consistência dos dados, as análises foram realizadas por meio de pacote estatístico Stata 13.1. A análise foi ponderada com base no sexo, na idade e educação formal dos indivíduos. Os pesos foram determinados pela razão entre as proporções de indivíduos na amostra do estudo.^21^ Foram calculadas as proporções, médias e respectivos intervalos de confiança das variáveis estudadas para a amostra geral e amostra estratificada por sexo, considerando nível de confiança de 95% (IC95%). A diferença estatística entre as amostras avaliadas foi verificada pela comparação de intervalos de confiança, sendo aqueles que não apresentaram sobreposição significativa. A variável dependente normal CMO foi analisada por meio do teste de Shapiro-Wilk e de histogramas, indicando distribuição normal. As associações entre o CMO e as variáveis independentes foram investigadas por regressão linear, utilizando p<0,25 como critério de entrada no modelo múltiplo, e nível de significância global de α=0,05.

Esta pesquisa foi aprovada pelo Comitê de Ética em Pesquisa da Universidade Federal de Viçosa (UFV) (02/2013/CEP/12.07.2013) conforme princípios da Declaração de Helsinque e da resolução 466/2012 do Conselho Nacional de Saúde. Todos os voluntários que concordaram em participar do estudo assinaram o Termo de Consentimento.

## RESULTADOS

Do total de 701 indivíduos incluídos no estudo, 50,3% eram mulheres, 26,2% tinham idade entre 30 e 39 anos, 61,4% identificaram-se como não brancos, e 42,8% tinham concluído mais de 12 anos de estudos. Na amostra, 50,3% dos indivíduos eram eutróficos, 72,6% dos indivíduos eram irregularmente ativo, 65,1% eram não fumantes e 45,9% não consumiam bebidas alcoólicas. Em relação à condição de saúde, 49,0% dos pacientes avaliados apresentaram suficiência de 25(OH)D. Do total de mulheres avaliadas, 64,7% eram não menopausadas, 96,4% não faziam reposição hormonal e 81,0% usavam contraceptivos. Em relação à comparação das proporções entre os sexos, 62,0% das mulheres não consumiam bebidas alcoólicas e 23,2% dos homens consumiam oito ou mais doses de bebida alcoólica. Por esse motivo, as diferenças nessas categorias foram significativas. Além disso, os homens apresentaram menor proporção de deficiências de 25(OH)D do que as mulheres, sendo essa diferença também foi significativa ( Tabela 1 ).

**Tabela 1:** Caracterização da população do estudo, de acordo com variáveis sociodemográficas, comportamentais e condições de saúde, estratificada por sexo

Variáveis	Total		Homens		Mulheres	
		
	%	IC95%	%	IC95%	%	IC95%
Sexo			49,7	45,2-54,2	50,3	45,8-54,8
Faixa etária, anos						
20-29	24,3	17,4-33,4	28,9	19,1-41,2	19,8	13,8-27,6
30-39	26,2	21,8-31,0	26,6	20,4-34,0	25,7	21,0-31,0
40-49	24,0	19,1-29,7	21,9	15,2-30,4	26,2	20,6-32,5
50-59	25,5	20,1-31,7	22,6	16,1-30,8	28,3	22,2-35,4
Cor da pele						
Branco	38,6	31,7-45,9	42,0	32,9-51,7	35,2	28,6-42,3
Não branco	61,4	54,1-68,3	58,0	48,3-67,1	64,8	57,7-71,3
Educação formal, anos						
0-4	20,1	12,8-29,1	16,7	7,7-32,8	23,4	16,2-32,5
5-8	16,3	11,9-21,7	15,7	10,4-23,0	16,7	11,9-23,0
9-11	20,8	17,4-24,7	20,0	14,1-27,3	21,7	18,7-25,1
≥12	42,8	30,8-55,7	47,6	32,6-63,1	38,2	27,1-50,5
Estado nutricional						
Eutrófico	50,3	43,2-57,4	47,7	38,7-56,8	53,0	43,5-62,2
Sobrepeso	33,4	28,3-38,8	39,6	31,6-48,2	27,2	22,1-33,0
Obesidade	16,3	12,7-20,5	12,7	8,8-17,9	19,8	14,6-26,1
NAF						
IA	72,6	66,8-78,4	73,13	63,5-80,9	72,16	65,9-77,6
FA	27,4	21,6-33,9	26,86	19,0-36,5	27,84	22,4-34,0
Tabagismo						
Não fumante	65,1	57,9-71,6	61,9	50,5-72,1	68,3	60,7-75,1
Fumante	13,0	9,9-16,5	15,2	9,8-22,7	10,7	7,8-14,7
Ex-fumante	21,9	15,8-29,5	22,9	13,5-36,3	21,0	15,3-28,0
Consumo de álcool, dose/semana						
0	45,9	41,7-50,2	29,8	23,8-36,6^*^	62,0	55,5-68,0^*^
1-7	40,3	35,1-45,7	47,0	38,1-55,0	33,7	28,1-39,8
8 ou mais	13,8	10,0-18,4	23,2	17,4-30,3^*^	4,3	2,5-7,5^*^
Menopausa					64,7	56,9-71,7
Não	-	-	-	-	35,3	28,3-43,1
Sim	-	-	-	-		
Terapia de reposição hormonal						
Não	-	-	-	-	96,4	94,3-97,7
Sim	-	-	-	-	3,6	2,3-5,7
Contraceptivo						
Não	-	-	-	-	19,0	15,4-23,1
Sim	-	-	-	-	81,0	76,8-84,6
25(OH)D						
Suficiente	49,0	42,5-55,5	55,7	47,4-63,6	42,4	35,0-50,0
Insuficiente	37,5	32,7-42,6	35,3	28,8-42,3	39,8	34,7-45,0
Deficiente	13,5	10,5-17,1	9,0	6,3-12,7^*^	17,8	13,1-23,8^*^

* Diferença estatisticamente significante, (p<0,05) para comparação entre sexo. IC95%: intervalo de confiança de 95%; NAF: nível de atividade física; IA: irregularmente ativo; FA: fisicamente ativo; 25(OH)D: 25-hidroxivitamina D.

Os valores médios de CMO foram significativamente maiores entre os homens. Foi observada melhor saúde óssea entre os indivíduos com sobrepeso e obesidade, comparados aos eutróficos de ambos os sexos. Um CMO médio mais elevado foi encontrado apenas entre mulheres mais jovens (grupo de 20 a 29 anos) comparado com mulheres mais velhas (grupo de 50 a 59 anos), que se autoidentificaram como não brancas, não menopausadas, e que não faziam uso de contraceptivos ( Tabela 2 ).

**Tabela 2:** Conteúdo mineral ósseo, de acordo com variáveis sociodemográficas, comportamentais e de condição de saúde, estratificadas por sexo

Variáveis	Homens		Mulheres	
	
	Média	IC95%	Média	IC95%
Sexo	3,040	2,972-3,109	2,323	2,284-2,362
Faixa etária, anos				
20-29	3,122	3,025-3,219	2,415	2,336-2,495^*^
30-39	3,042	2,929-3,154	2,408	2,331-2,486
40-49	3,031	2,894-3,169	2,359	2,297-2,421
50-59	2,944	2,816-3,073	2,148	2,050-2,247^*^
Cor da pele				
Branca	2,985	2,895-3,075	2,245	2,187-2,302^*^
Não branca	3,081	2,976-3,186	2,365	2,306-2,425^*^
Educação (anos)				
0-4	2,878	2,716-3,040	2,223	2,102-2,345
5-8	2,968	2,830-3,106	2,373	2,265-2,480
9-11	3,094	2,950-3,238	2,312	2,230-2,394
≥12	3,101	3,006-3,196	2,369	2,313-2,425
Estado nutricional				
Eutrófico	2,835	2,744-2,927*^†^	2,225	2,183-2,267*^†^
Sobrepeso	3,175	3,043-3,307*	2,396	2,313-2,479*
Obesidade	3,423	3,307-3,539^†^	2,499	2,388-2,609^†^
NAF				
IA	2,992	2,923-3,060	2,319	2,268-2,369
FA	3,174	3,032-3,316	2,333	2,264-2,401
Tabagismo				
Não fumante	3,041	2,952-3,130	2,328	2,285-2,371
Fumante	3,105	2,994-3,217	2,302	2,190-2,414
Ex-fumante	2,996	2,894-3,098	2,317	2,224-2,410
Consumo de álcool, doses/semana				
0	2,999	2,866-3,133	2,319	2,270-2,368
1-7	3,038	2,945-3,131	2,322	2,251-2,393
8 ou mais	3,098	2,953-3,243	2,390	2,195-2,586
Menopausa				
Não	-	-	2,396	2,336-2,455
Sim	-	-	2,190	2,103-2,277
Terapia de reposição hormonal				
Não	-	-	2,323	2,283-2,363
Sim	-	-	2,309	2,152-2,466
Contraceptivo				
Não	-	-	2,294	2,243-2,346^*^
Sim	-	-	2,429	2,346-2,513^*^
25(OH)D				
Suficiente	3,100	3,019-3,181	2,339	2,283-2,395
Insuficiente	2,994	2,874-3,113	2,298	2,241-2,356
Deficiente	2,861	2,693-3,028	2,338	2,229-2,446

*^†^Símbolos iguais indicam diferença estatisticamente significante (p<0,05) para a comparação entre as médias de conteúdo mineral ósseo segundo as categorias das variáveis dentro de cada sexo. IC95%: intervalo de confiança de 95%a; NAF: nível de atividade física; IA: irregularmente ativo; FA: Fisicamente ativo; 25(OH)D: 25-hidroxivitamina D.

As tabelas 3 e 4 mostram associações não ajustadas e ajustadas entre o CMO e variáveis independentes, de acordo com o sexo. O CMO diminui com a idade em ambos os sexos (homens: p=0,008; mulheres: p<0,001), e esta associação foi mais pronunciada após o ajuste (homens: p=0,007; mulheres: p<0,001). A variável estado nutricional também foi associada à CMO nos modelos ajustados para ambos os sexos (homens: p<0,001; mulheres: p<0,001), constituindo uma associação positiva. Observou-se associação significativa entre o alto nível de educação formal e o aumento do CMO observado apenas entre homens (p=0,003) e deficiência de 25(OH) D que se associou significativamente ao baixo CMO (p<0,001). O CMO também foi significativamente maior entre as mulheres que se autoidentificaram não brancas (p=0,010).

**Tabela 3:** Coeficientes de regressões lineares simples e múltiplos, intervalos de confiança e valor de p para o conteúdo mineral ósseo nos homens

CMO (g)	β	IC95%	Valor de p*	βaj	IC95%	Valor de p^†^
Faixa etária, anos			0,008			0,007
20-29	-	-		-	-	
30-39	-0,08	-0,22-0,06		-0,03	-0,16-0,08	
40-49	-0,09	-0,24-0,06		-0,14	-0,28- -0,06	
50-59	-0,17	-0,31- -0,03		-0,17	-0,31- -0,03	
Educação formal, anos			0,011			0,003
0-4	-	-				
5-8	0,09	-0,12-0,30		0,11	-0,11-0,33	
9-11	0,21	-0,02-0,45		0,20	-0,03-0,43	
≥12	0,22	0,03-0,41		0,27	0,07-0,48	
Estado nutricional			<0,001			<0,001
Eutrófico	-	-		-	-	
Sobrepeso	0,33	0,18-0,49		0,41	0,28-0,54	
Obesidade	0,58	0,43-0,74		0,67	0,5-0,85	
25(OH)D			0,005			<0,001
Suficiente	-	-		-	-	
Insuficiente	-0,10	-0,23-0,02		-0,14	-0,26 -0,02	
Deficiente	-0,23	-0,4- -0,07		-0,32	-0,45- -0,18	

* Regressão linear simples com nível de significância de p<0,25;^†^regressão linear múltipla com nível de significância de p<0,05. β: regressão linear simples; IC95%: intervalo de confiança de 95%; βAj: β adjustado para covariâncias; CMO: conteúdo mineral ósseo; 25(OH)D: 25 hidroxivitamina D.

**Tabela 4:** Coeficientes de regressões lineares simples e múltiplos, intervalos de confiança e valor de p para o conteúdo mineral ósseo em mulheres

CMO (g)	β	IC95%	Valor de p*	βaj	IC95%	Valor de p^†^
Faixa etária, anos			<0,001			<0,001
20-29	-	-		-	-	
30-39	-0,07	-0,10-0,90		-0,08	-0,18- 0,13	
40-49	-0,05	-0,16-0,05		-0,17	-0,27 -0,65	
50-59	-0,02	-0,04-0,01		-0,38	-0,53- -0,24	
Cor da pele			0,013			0,010
Branco	-	-				
Não Branco	0,12	0,02-0,21		0,91	0,24-0,15	
Estado nutricional			<0,001			<0,001
Eutrófico	-	-		-	-	
Sobrepeso	0,17	0,08-0,25		0,22	0,14-0,31	
Obesidade	0,73	0,16-0,17		0,34	0,26-0,43	

* Regressão linear simples com nível de significância p<0,25;^†^regressão linear múltipla com nível de significância p<0,05. β: regressão linear simples; IC95%: intervalo de confiança de 95%; βAj: β adjustado para covariâncias; CMO: conteúdo mineral ósseo; 25(OH)D: 25 hidroxivitamina D.

## DISCUSSÃO

Em nosso estudo, o CMO médio nos homens foi significativamente maior que entre as mulheres. Além disso, em relação à diferença entre os sexos, os principais fatores associados ao CMO foram idade e estado nutricional. Outra observação foi a associação entre educação formal e 25(OH)D entre os homens. Nas mulheres, esta associação esteve relacionada com a cor da pele.

Nossos achados sobre a associação entre sexo e saúde óssea para mulheres apresentaram CMO média significativamente menor, o que é consistente com outros estudos.^3 , 4^ Essa diferença está relacionada principalmente à problemas hormonais^29^ e se intensifica após a menopausa, quando há redução significativa dos níveis de estrogênio.

A perda de CMO durante a vida adulta também pode estar relacionada a fatores ambientais^30^ e genéticos,^5^ bem como ao uso prolongado de contraceptivos.^18^

Nossos resultados, em relação à idade, corroboram outros estudos.^3 , 4^ Identificou-se que, à medida que a idade aumenta, o equilíbrio entre a formação e a absorção óssea alterado, e o CMO diminui progressivamente. Essa perda é mais pronunciada após o pico de massa óssea, que ocorre por volta dos 30 anos, e então se reduz gradualmente, intensificando-se com o avanço da idade.^31^ Portanto, o metabolismo ósseo balanceado, durante a vida adulta, proporciona manutenção adequada à saúde óssea e pode ser fator importante no controle da perda de CMO e para se evitar o risco de fraturas em indivíduos mais velhos.

Após o período de pico de massa óssea procedimentos preventivos devem ser iniciados para evitar declínio ósseo acentuado em idades mais avançadas.

Nesse sentido, a atividade física regular é fator importante, que pode atuar como alternativa não farmacológica para prevenir as consequências decorrentes do baixa massa óssea.^14^ Apesar de não terem sido observadas diferenças significativas relacionadas com essa variável em nosso estudo, um CMO médio elevado foi encontrado entre os indivíduos fisicamente ativo. Assim, são necessárias mais investigações relacionando o CMO e o histórico dos indivíduos em relação ao NAF durante todo seu desenvolvimento.

Neste estudo, o estado nutricional (sobrepeso e obesidade) foi fator positivamente associado ao CMO, tanto nos homens quanto nas mulheres. Esse resultado é consistente com achados relatados por outro estudo com adultos jovens,^10^ e essa associação positiva pode ser explicada pela maior sobrecarga mecânica entre indivíduos com excesso de peso. Tais achados reforçam a teoria de que o tecido ósseo precisa sofrer estresse mecânico para a formação e remodelação óssea, mantendo o metabolismo ósseo ativo.^11^ Por outro lado, deve-se reforçar que existem consequências hormonais de alto peso corporal, que podem prejudicar a saúde óssea, como diabetes tipo 2, síndrome metabólica, resistência à insulina e hiperglicemia, entre outras.^32^

As associações positivas entre nível de educação formal e CMO também foram identificadas entre os homens, indicando que quanto maior o tempo de estudo, melhor a saúde óssea. Esses dados corroboram os de outros estudos que incluíram adultos.^15 , 16^ Essa associação positiva supõe que, para doenças relacionadas à saúde óssea, quanto maior o nível de escolaridade, maiores o acesso à informação e o conhecimento sobre cuidados preventivos e atitudes, como prática de atividade física regular e dieta equilibrada, o que pode refletir positivamente na saúde óssea de indivíduos com idade mais avançada.^15 , 16^

Outro achado do nosso estudo foi a relação direta entre a suficiência de 25(OH)D e o CMO entre os homens. Resultados semelhantes foram relatados em outros estudos.^8 , 9^ Dada a importância da 25(OH)D para o metabolismo ósseo (absorção e manutenção dos níveis séricos de cálcio e fosfato),^27^ se, durante a idade adulta, o metabolismo desta vitamina estiver equilibrado, pode-se manter a saúde óssea adequada nesse estágio de desenvolvimento, e este pode ser fator importante para controlar a perda de CMO e evitar o risco de fratura em indivíduos com idade mais avançada.

Identificamos a cor da pele como fator de associação para as mulheres, estando as mulheres não brancas positivamente associadas ao CMO. Existe grande variação nas concentrações de 25(OH)D de acordo com a cor da pele. Conforme relatado por outros estudos, indivíduos não brancos apresentam 25(OH)D insuficiente, mas apresentam melhor saúde óssea e menor risco para fratura.^33^

Esses achados requerem mais estudos, pois ainda existem lacunas na literatura quanto à associação entre cor da pele, metabolismo de 25(OH)D e saúde óssea nas diferentes populações.

Os aspectos positivos deste estudo são sua abordagem de base populacional, o uso de instrumentos validados, a padronização de procedimentos de coleta de dados, e a adoção de estratégias de controle de qualidade. Além disso, nosso estudo avaliou os fatores de risco para saúde óssea reduzida em homens saudáveis, o que é diferente da maioria dos estudos que avaliaram mulheres no período pós-menopausa, indivíduos com idade avançada e a relação desses grupos com doença específica.

Considerando que o pico de massa óssea ocorre por volta dos 30 anos em ambos os sexos e que um declínio gradual ocorre posteriormente, também avaliamos se os fatores associados à CMO diferem em indivíduos mais jovens comparados com aqueles com mais de 30 anos. No entanto, não foram observadas mudanças significativas em relação à magnitude, direção e significância estatística das associações nas análises estratificadas.

Outra questão considerada em nossa análise foi a possível limitação do IMC como método para avaliar o sobrepeso e a obesidade. Por esse motivo, ao introduzir a percentagem de gordura, consideramos as categorias do modelo final, com base no protocolo triplo,^34 , 35^ que substituiu a variável IMC. Nessa análise, identificamos que o modelo final permaneceu inalterado, e os resultados apresentaram os mesmos fatores de associação, tanto para homens quanto para mulheres. Nosso estudo avaliou os fatores associados ao CMO, e os resultados são apresentados pelo IMC, por ser uma medida prática recomendada pela OMS, de fácil aplicação e de baixo custo^26^ para classificação do estado nutricional em indivíduos saudáveis com sobrepeso ou baixo peso.

Este é um estudo transversal e, como tal, as associações identificadas não podem ser interpretadas como relação causal. Nossos resultados podem contribuir para melhor compreender a saúde óssea em adultos, bem como para o desenvolvimento de estratégias de prevenção relacionadas à saúde óssea.

## CONCLUSÃO

Os achados deste estudo indicam que os principais fatores associados ao baixo conteúdo mineral ósseo foram idade avançada, cor da pele branca, baixa escolaridade, eutrofia e deficiência de 25(OH)D. Diferenças foram observadas entre homens e mulheres. Indivíduos adultos com tais características devem ser considerados e aconselhados sobre as estratégias de prevenção e diagnóstico precoce. Outros estudos são necessários para aprofundar os entendimentos sobre os parâmetros comportamentais, como nível de atividade física, tabagismo e alcoolismo, pois a literatura ainda é escassa quanto à associação desses parâmetros com a saúde óssea.
